# A Rare Case of Trifoliate Gallbladder in an Adult Male and Its Management: A Comprehensive Case Report and Literature Review

**DOI:** 10.7759/cureus.47180

**Published:** 2023-10-17

**Authors:** Sayantan Patra, Soumya Suvra Patra

**Affiliations:** 1 Interventional Radiology, Rabindranath Tagore International Institute of Cardiac Sciences, Kolkata, IND; 2 Radiology, Vardhman Mahavir Medical College (VMMC) & Safdarjung Hospital, New Delhi, IND; 3 Internal Medicine, Calcutta National Medical College, Kolkata, IND

**Keywords:** murphy's sign, trifoliate gallbladder type 3, maximum intensity projection (mip), magnetic resonance cholangiopancreatography (mrcp), triple gallbladder

## Abstract

Triple gallbladder, a rare congenital abnormality resulting from the incomplete regression of rudimentary bile ducts, often goes undetected until incidentally discovered during imaging studies. This report presents the case of a 38-year-old male with intermittent abdominal pain, food intolerance, and nausea. Physical examination findings included tenderness in the right hypochondrium and a positive Murphy's sign. Laboratory tests revealed leukocytosis and elevated liver enzymes, while abdominal ultrasound identified three fluid-filled sacs in the expected gallbladder fossa location, with one showing cholelithiasis and mild wall thickening in all three. Further evaluation via magnetic resonance cholangiopancreatography (MRCP) unveiled three incompletely separated tubular cystic structures draining into a solitary cystic duct, confirming the diagnosis as trifoliate gallbladder type 3. This case highlights the importance of considering rare entities like triple gallbladder in the differential diagnosis of biliary pathologies, necessitating a high index of suspicion and comprehensive imaging to ensure accurate diagnosis and appropriate management, as exemplified by successful laparoscopic cholecystectomy.

## Introduction

Vesica fellae triplex, often known as a triple gallbladder, is a very rare congenital malformation of the biliary system [1]. The available preoperative imaging methods were fairly constrained because the bulk of previously reported triple gallbladder instances (9 out of 10) were reported between 1926 and 1979. These methods included tomography, fat meal studies, oral or intravenous cholecystogram, and postoperative T-tube cholangiography [2, 3]. Preoperative tests include sonography and endoscopic retrograde cholangiopancreatography, which, in the most recent instance described in 2003, failed to find any anatomical abnormalities in the biliary system. Instead, the diagnosis was made prior to surgery for a few cases [4]. According to earlier accounts, the clinical importance of finding a double or triple gallbladder is that there is a higher chance that these additional gallbladders contain diseased cells. Sludge, gallstones, inflammation (cholecystitis), cellular alterations (metaplasia), and in some extremely rare cases, even cancer, may all be included in these diseases [5, 6]. Additionally, knowing about this anatomical variance before surgery can considerably lower the likelihood of unanticipated difficulties during cholecystectomy and avoid unintentional injury to the biliary system. Additionally, it is crucial that surgeons be aware of this abnormality beforehand to guarantee that all gallbladders are removed, avoiding the development of "postcholecystectomy syndrome" brought on by the retention of an auxiliary gallbladder. We present the case of a 38-year-old male who presented with abdominal pain, elevated liver enzymes, and leukocytosis. Imaging revealed trifoliate gallbladder type 3, confirmed with magnetic resonance cholangiopancreatography (MRCP). A laparoscopic cholecystectomy was performed without complications.

## Case presentation

We present the case of a 38-year-old male who presented to the emergency department with a history of intermittent, colicky abdominal pain, food intolerance, bloating, nausea, and vomiting for the past three days. No significant history of asthma, diabetes mellitus, or tuberculosis was found.

On physical examination, tenderness in the right hypochondrium and a positive Murphy’s sign on deep palpation were elicited. No rigidity or rebound tenderness could be elicited. A complete blood count (CBC) revealed leukocytosis, while biochemical analyses revealed elevated transaminase levels: alanine transaminase (ALT), aspartate transaminase (AST), and alkaline phosphatase (ALP). Total bilirubin was found to be within the normal range, and amylase and lipase levels were also reported. Other routine biochemistries were normal, as shown in Table 1.

**Table 1:** Hematological and biochemical parameters of the patient.

Laboratory Test	Result	Reference Range
Complete Blood Count (CBC)		
- Leukocyte Count	12,200/mL	Normal: 4,000-11,000/mL
Biochemistries		
- Alanine Transaminase (ALT)	312 U/L	Normal: 7-56 U/L
- Aspartate Transaminase (AST)	184 U/L	Normal: 15-37 U/L
- Alkaline Phosphatase (ALP)	186 U/L	Normal: 30-120 U/L
- Total Bilirubin	0.9 mg/dL	Normal: 0.2-1.2 mg/dL
- Amylase	40 U/L	Normal: 30-110 U/L
- Lipase	22 U/L	Normal: 10-140 U/L

**Figure 1:**
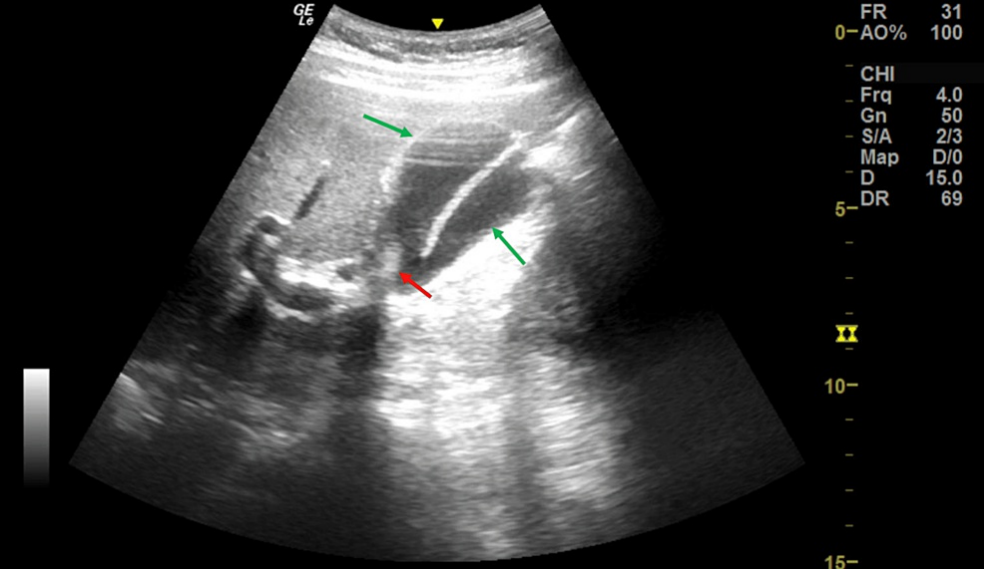
Lateral sagittal section ultrasound image shows two anechoic, fluid-filled, saccular structures (green arrows) in GB fossa and a shadowing calculus at the common neck (red arrow).

Given the raised leucocyte count and transaminase, an abdominal ultrasound was requested to the radiology department. Figure 1 is an image of the ultrasound investigation depicting two fluid-filled sacs that were not echoing and were in the GB fossa. The walls of all three structures were slightly thickened. Sonographic results suggested either a Todani type II choledochal cyst or a second gallbladder, and a magnetic resonance cholangiopancreatography (MRCP) was recommended.

**Figure 2:**
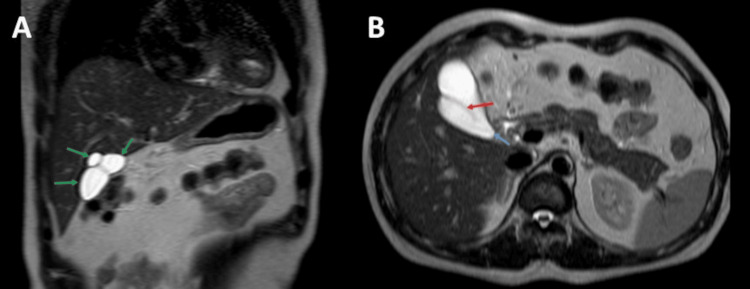
Sagittal section T2W image reveals three separated tubular, cystic structures in the gallbladder fossa with intervening septations (green arrows) (A). Trans-axial section T2W image shows incomplete septation (red arrow) between two gallbladders sharing a common neck (blue arrow) (B).

MRCP showed the presence of three incompletely separated tubular, cystic structures in the region of gallbladder fossa with incomplete septations on multiple views as shown in Figure 2.

**Figure 3:**
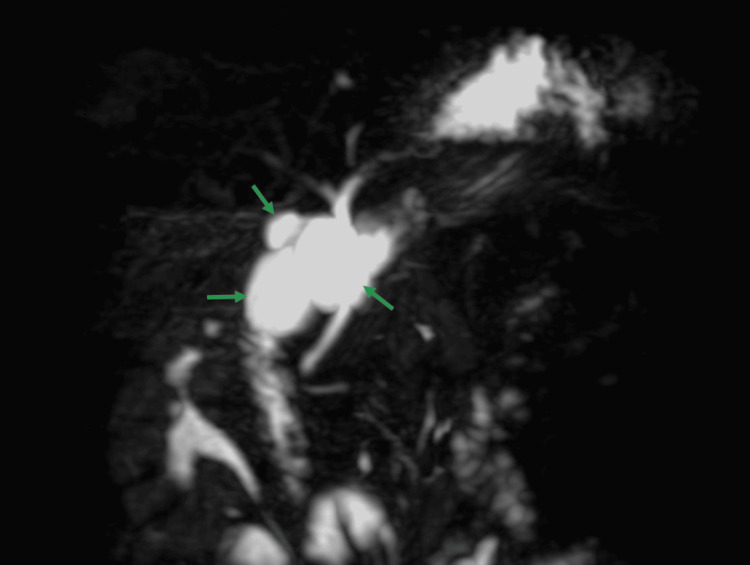
Maximum Intensity Projection (MIP) showing triplication of gallbladder (green arrows) with three separate lumens.

The structures observed in the imaging confluenced at the gallbladder's neck, connected by a single cystic duct. These findings align with the diagnosis of trifoliate gallbladder type 3, which was further confirmed through a Maximum Intensity Projection (MIP) image displayed in Figure 3.

**Figure 4:**
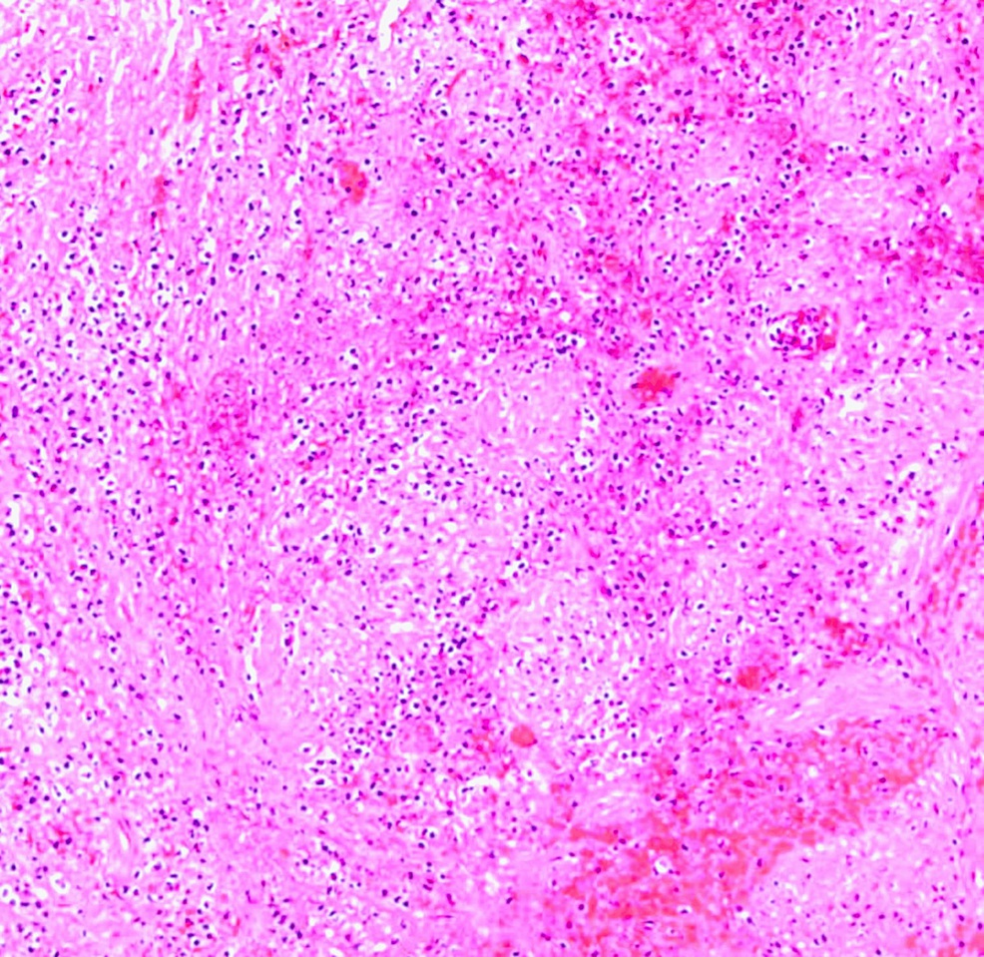
Histopathological examination (HPE) image with haematoxylin-eosin stain showing fibrinous, inflammatory exudates and neutrophil infiltration into the gallbladder mucosa consistent with acute cholecystitis.

Following appropriate informed consent, the patient was taken for laparoscopic cholecystectomy to treat the calculous cholecystitis. Laparoscopic exploration confirmed the presence of triplication of gallbladder lumen with a single cystic duct and an accompanying artery. The surgical specimen was sent for histopathological examination which revealed slightly edematous mucosa in two of the three gallbladders and slightly edematous, inflammatory changes in the mucosa of the third with accompanying calculus. No signs of chronic inflammation, metaplasia or carcinomatous changes were noted in any of the three gallbladders, as shown in Figure 4. The post-operative recovery was uneventful. The patient responded well to the treatment given and was later discharged and scheduled for further outpatient follow-up.

## Discussion

The persistence of primitive bile ducts that do not undergo regression throughout embryological development is thought to be the cause of many gallbladders. This abnormality can develop over time as a result of these recurring cell outgrowths along the common bile duct. The origin of these persistent rudimentary duct buds appears to define the relationship of the gallbladder to the cystic and common bile ducts. The gallbladders will have distinct cystic ducts, whether these buds come from the hepatic duct or the common bile duct. On the other hand, the gallbladders will share a single common cystic duct that empties into a regularly arranged common bile duct if these buds come from the cystic duct itself. Based on the unique anatomical design of the cystic ducts, three different varieties are distinguished.

**Figure 5:**
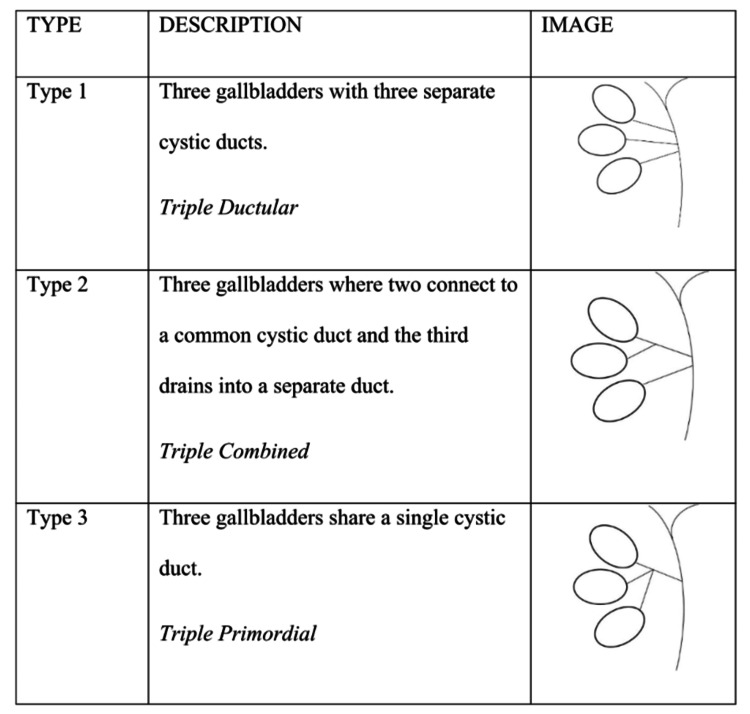
Types of Trifoliate gallbladder

The primary characteristic of this kind is the presence of several gallbladders, each of which has a distinct cystic duct connecting it to the main bile duct. The second kind has a third gallbladder with its own independent cystic duct in addition to two gallbladders with a shared cystic duct entering the common duct. In the third form, there are three gallbladders present, and they all share a single cystic duct as shown in Figure 5 [2, 3]. Gallstones (cholelithiasis), sludge buildup, inflammation (cholecystitis), cellular alterations (cellular metaplasia), and even cancer have all been linked to having multiple gallbladders. Roeder et al., for instance, described a case in which one gallbladder had cholecystitis with gallstones, whereas the other contained a papillary adenocarcinoma [6].

In modern medicine, biliary imaging relies on cutting-edge, high-resolution procedures like magnetic resonance cholangiopancreatography (MRCP), spiral or multislice computed tomography (CT), and endoscopic retrograde cholangiopancreatography (ERCP). With just one known exception in the pertinent literature, most recent instances of triple gallbladders have been difficult to identify prior to surgery for acute cholecystitis [5, 7].

The third lumen was misinterpreted as either a convolution inside one of the cystic ducts or a gallbladder abnormality, such as a partial transverse septum, which may have made it difficult to make a conclusive preoperative diagnosis. While CT and ultrasonography can show details like wall thickness, lumen abnormalities, and the number of gallbladders, they are insufficient for fully defining the complex biliary tree architecture [8].

It is advised to supplement these techniques with either MRCP or ERCP in order to fully display the architecture and any changes within the biliary system. It's crucial to remember that, in some circumstances, even the most extensive combinations of imaging modalities may not be able to provide a precise diagnosis. It is advised to choose to have all of the gallbladders removed if any of them show symptoms in order to avoid future problems and the potential need for additional surgery [1]. There are several procedures for removing a triple gallbladder that have been described in the literature [9]. Due to the many anatomical configurations seen, some specialists support open cholecystectomy [10]. However, it is advised to do intraoperative cholangiography for direction if a laparoscopic method is chosen.

## Conclusions

In conclusion, the presented case of triple gallbladder, a rare congenital anomaly of the biliary system, highlights the importance of thorough preoperative evaluation and awareness among clinicians. The diagnosis of this condition can be challenging, and it often requires a combination of advanced imaging modalities such as MRCP or ERCP to accurately define the complex biliary architecture. Recognizing this anatomical variation before surgery is crucial to preventing unexpected complications during cholecystectomy and ensuring the complete removal of all gallbladders, reducing the risk of "postcholecystectomy syndrome". Additionally, the clinical significance of triple gallbladders lies in their association with various biliary pathologies, emphasizing the importance of their early detection and management. This case underscores the need for a high index of suspicion and the utilization of multiple imaging techniques for an accurate diagnosis and successful surgical intervention when necessary.
